# Dorsal wrist ganglion: clinical and imaging correlation in symptomatic population based on high-field MRI

**DOI:** 10.1007/s00330-024-10831-3

**Published:** 2024-06-10

**Authors:** David Ferreira Branco, Paul Botti, Cindy Bouvet, Bilal Abs, Marcello Buzzi, Jean Yves Beaulieu, Pierre-Alexandre Poletti, Hicham Bouredoucen, Sana Boudabbous

**Affiliations:** 1 Diagnostic Department, Radiology Unit, Geneva University Hospital, Rue Gabrielle-Perret-Gentil 4, 1205 Genève, Switzerland; 2 Orthopedic and Traumatology Surgery Department, Hand Surgery Unit, Sion Hospital, Av. du Grand-Champsec 80, 1951 Sion, Switzerland

**Keywords:** Wrist, Dorsal cyst, Magnetic resonance imaging, Epidemiology

## Abstract

**Objectives:**

To determine prevalence in the symptomatic population of dorsal mucoid cysts centered on dorsal capsuloscapholunate septum (DCSS) using high-field magnetic resonance imaging (MRI) for anatomoclinical and epidemiological correlations.

**Materials and methods:**

This single-center retrospective study analyzed all 3-Tesla MRIs consecutively performed for painful wrists in 295 patients. Two blinded readers performed measurements. The protocol included T1 spin echo and 3D proton density sequences with fat saturation. Inter-observer reliability was assessed using kappa and intra-class correlation coefficients for cyst detection and volumetry, respectively. Disagreements concerning cyst detection were resolved by a consensus reading. Cyst size, relationship to extrinsic and scapholunate ligaments (SL), continuity of SL, minimum distance to the posterior interosseous nerve (PIN), cyst communication with joint, and anatomical classifications of cysts were analyzed. Correlation tests were performed to assess associations.

**Results:**

Two-hundred ninety-five patients (mean age 39.6 +/− 15.6 (standard deviation), 161 males) were evaluated for detection of dorsal wrist cysts identified in 150/295. In this subgroup, the mean age was 38.7 years (15–75), the sex ratio of 0.6 (59% women), and the median volume cyst of 8.7 mm^3^ (0.52–2555). Cyst detection, volume, and major axis measurements showed very high agreement between observers, respectively, 0.89, 0.96, and 0.91. 42 patients had dorsal SL pain. A weak negative correlation was found between distance to PIN and dorsal SL pain (*r* = −0.2415; *p* < 0.05) and a weak positive correlation between Guérini’s classification and dorsal SL pain (*r* = 0.2466; *p* < 0.05).

**Conclusion:**

High-field MRI is the modality of choice for the detection, anatomical, and volumetric assessment of dorsal cysts. Preoperative assessment will be aided by the proposed revised anatomical classification.

**Clinical relevance statement:**

High-field MRI is the modality of choice for the anatomical study of dorsal ganglion cysts. It allows the radiologist to accurately describe the anatomical relationships, size, and visibility of the pedicle, essential information for the surgeon’s preoperative assessment.

**Key Points:**

- *Dorsal mucoid wrist ganglion is a condition for which prevalence remains to be determined.*
- *High-field MRI is a reproducible imaging modality for the detection and assessment of dorsal wrist cysts.*
- *High-field MRI has a key role in the preoperative management of dorsal mucoid cysts.*

## Introduction

**Fig. 1:**
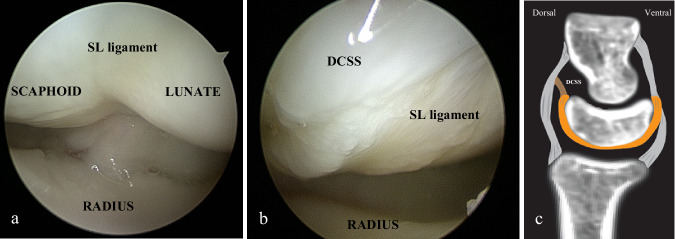
**a** Arthroscopic view of the radio-carpal joint (dorsal approach between the 3rd and 4th extensor compartments; portal 3-4). The scapholunate ligament is tight between the scaphoid and the lunate. It appears pearly white with a regular surface; **b** Arthroscopic view of the radio-carpal joint (portal 3-4). Between the scapholunate ligament and the dorsal joint capsule, we can observe the DCSS. **c** Anatomical illustration in sagittal view of the three components of the dorsal SL complex, with the dorsal part of the SL ligament in orange, the DIC (dorsal intercarpal ligament) integrating the dorsal capsule in gray, and the DCSS between these structures in brown

Ganglia are the most common benign soft tissue tumor of the hand [1] and those on the dorsal surface of the wrist represent 60 to 70% of wrist cysts [2, 3]. Their incidence is probably underestimated, as they are often neglected and, for the most part, asymptomatic. However, they can be a reason for consultation and treatment in rare cases where they are painful or for esthetic reasons [3]. The pathophysiology of these cysts has been debated for many years. Although initially, the oldest theory was that of a synovial hernia, other authors have suggested the possibility of a communication channel through the scapholunate ligament (SL) linking the joint cavity and the cyst, with a non-return valve mechanism [1, 2, 4]. Anatomical advances in the dorsal scapholunate region led to the description of the dorsal capsular scapholunate septum (DCSS) by Van Overstraeten [5, 6] (see Fig. 1), a fibrous structure derived from the dorsal capsule and inserted in a bifid way at the bone-ligament junction on the dorsal and superior aspect of the scapholunate joint, on the surface of the SL ligament, acting as a secondary stabilizer of SL joint [6]. This new anatomical knowledge combined with electron microscopy of the cyst wall confirming the absence of synovial cells and biochemical analysis of the mucoid fluid supporting its nature, have called into question its arthro-synovial nature [7]. The systematic origin [7] of the cyst in the deep DCSS in contact with the SL supports the “capsular rent” theory [2] as a consequence of joint stress causing leakage of synovial fluid towards the dorsal scapholunate complex resulting in the creation of gelatinous fluid and the formation of cyst walls by degeneration of mesenchymal cells.

The aim of this study was to determine the prevalence in the symptomatic population of the dorsal mucoid ganglion in the dorsal scapholunate region using 3-Tesla magnetic resonance imaging (MRI) for anatomoclinical and epidemiological correlations.

## Material and methods

### Patient population

In this retrospective study, we analyzed all consecutive 3-Tesla MRIs performed for painful wrists in 295 patients aged from 11 to 77 years (mean age of 35.6 years), between January 2019 to October 2023 in Geneva University Hospitals. The protocol was approved by the institutional ethical committee (Commission Cantonale de Recherche (CCR) number: 2017–01276). Exclusion criteria included contraindications to MRI *n* = 13; Inflammatory wrist disease *n* = 46; known or clinically suspected dorsal cyst *n* = 8; prior wrist surgery *n* = 11; cinetic artifact on 3D sequences *n* = 4.

### Imaging protocol and analysis

**Fig. 2:**
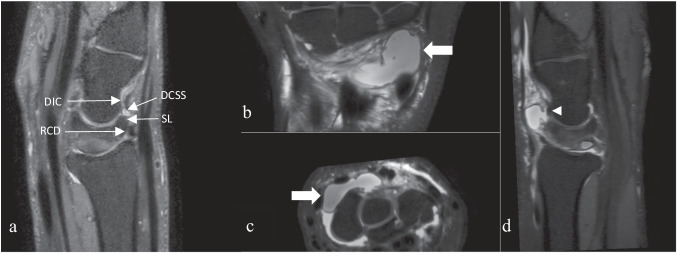
**a** Sagittal reconstruction of 3D DP SPAIR. DIC, dorsal intercarpal ligament; RCD, radio-carpal ligament; DCSS, dorsal capsular scapholunate septum with a ganglion inside; SL, scapholunate ligament. Images of large dorsal wrist ganglion using 3D DP SPAIR in MPR mode; **b** coronal plane; **c** axial plane; **d** sagittal plane. Arrow = ganglion; arrowhead = scapholunate ligament

Image analysis included coronal and axial T1 turbo spin echo (TSE), 3D Proton Density (PD) Fat Sat sequences (see Table 1) obtained in dStream HandWrist 16ch coil with arm abduction at 90°, elbow flexed at 45° and palm facing down on MRI 3.0 Tesla Phillips. 3D PD Fat Sat sequences were reconstructed in 3D planes using multiplanar reconstruction (MPR) mode (see Fig. 2) and images were digitally stored in a picture archiving and communication system (OsirixR, Pixmeo SARL, Bernex, Switzerland) under anonymized conditions. Cases were anonymized and randomized. In order to avoid inter-individual variability in measurements, a consensus reading was carried out for five cases 1 month before the start of the study. These cases were anonymized and randomized in the study to include them in statistical measurements. The criteria for diagnosis of ganglion were: single or multiloculated spherical and/or ellipsoidal well-demarcated mass with PD fat sat fluid hypersignal, T1 hyposignal arising in the dorsal scapholunate region with or without a direct relationship with the SL ligament but centered on the DCSS. Two radiologists specialized in osteoarticular disorders with 6 years (D.F.B.) and 5 years of experience (P.B.), respectively, accomplished the measurements separately. In order to minimize the observer variability, we applied a standardized protocol for data recording, and images were analyzed for parameters such as the presence or absence of a cyst, its size (volume calculation for spherical cyst *V* = 4/3 π r³, r: radius; for ellipsoid cyst *V* = 4/3 π abc, a:length b:width; c:height), location, relationship to the extrinsic ligaments and the SL, as well as the continuity of the latter (classified as discontinuous if partial or complete tear of dorsal ligament), the minimum distance to the posterior interosseous nerve (PIN) in relation to the cyst, cyst communication with the joint, grading of the cyst according to the classification proposed by Guérini [8] and grading according to a classification modified by us (see “Discussion”). Cases where observers disagreed on the detection of a cyst were submitted to a consensus reading *n* = 15. Images were correlated with the patient’s symptoms, in particular the presence or absence of dorsal pain in the wrist, with epidemiological data such as age, sex, occupation, dominant hand, hand affected by the cyst, and the occurrence of associated acute trauma through retrospective analysis of the patient’s medical records, in particular the hand surgeon’s consultation. No patient had to be excluded for lack of information.

**Table 1:** Sequences parameters on 3-T MRI Phillips

Parameters	3D DP SPAIR	T1 TSE coronal	T1 TSE axial
FOV	110 × 110 mm	100 × 100 mm	80 × 100 mm
Slice thickness	0.45 mm	2.5 mm	2.5 mm
Interslice gap	0 mm	0.25 mm	0.25 mm
Matrix	316 × 268	500 × 397	268 × 247
Voxel size	0.2 × 0.2 mm	0.18 × 0.18 mm	0.16 × 0.16 mm
TR	1400	663	694
TE	32	15	10
Number of acquisitions	1.5	1	1.4
Parallel imaging Acceleration factor	CS 6	none	2
Acquisition time	5 min 33	3 min 36	2 min 52

### Statistical analysis

In addition to descriptive statistics, to estimate inter-observer reliability values, Kappa for binary or ordinary qualitative variables and intraclass correlation coefficients for quantitative continuous variables were used with Bland-Altman plots for illustration purposes. Owing to Gaussian distribution, a paired *t*-test was applied to compare the findings between observers. For the interpretation of agreement statistic scores, we used the criteria developed by Landis and Koch [9]. Based on these criteria, values of 0–0.20 represent slight agreement, 0.21–0.40 fair agreement, 0.41–0.60 moderate agreement, 0.61–0.80 substantial agreement, and 0.81–1 almost perfect agreement. Pearson correlation tests were also carried out to assess the strength of association between the other variables measured. Statistics were performed on MedCalc software (Ostend Belgium).

## Results

**Fig. 3:**
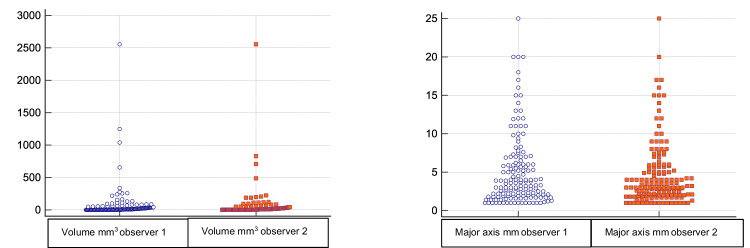
Schematic distribution for ganglion volume and major axis for both readers

**Fig. 4:**
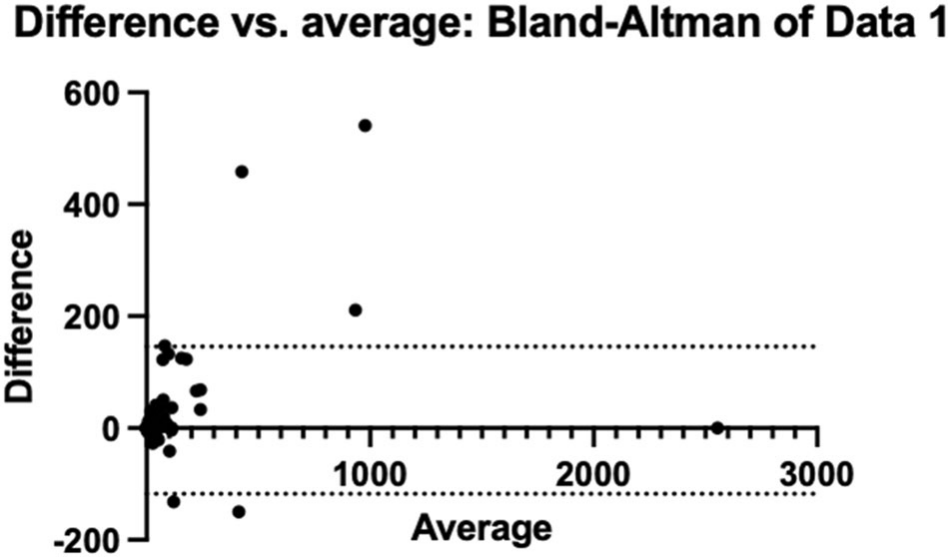
Bland-Altman plots illustrate differences in volume measurements in mm^3^ between observers according to the volume of cysts studied

The descriptive statistics of our study population are summarized in Table 2. Inter-observer agreement for cyst detection was almost perfect (weighted Kappa: 0.89; 95% CI: 0.84 to 0.94). Of the 295 wrists studied on imaging, 50.8% (*n* = 150) showed a dorsal wrist cyst centered on the DCSS and the deep part of the dorsal SL. In this population of patients with cysts, the mean age was 38.7 years (aged 15 to 75 years), and 50.6% (*n* = 76) of patients had an acute trauma requiring examination. The sex ratio was 0.6 with 58.7% women (88/150) and 41.3% men (62/150). 88% of patients were right-handed (*n* = 132) and 49.3% (*n* = 74) of wrists had a ganglion on the left side, showing an equitable distribution between the two sides. The median cyst volume was 8.7 mm^3^ and 8.2 mm^3^ for observers 1 and 2, respectively (minimum: 0.52 mm^3^; maximum 2555 mm^3^) with the median major axis at 3 mm ranging from 1 to 25 mm for both observers. Most cysts were small, with a 75th percentile volume and major axis of 35.5 mm^3^ and 6.3 mm, respectively (see Fig. 3). Volume and major axis cysts measurements displayed very high agreement between observers of 0.96 (95% confidence interval 0.95 to 0.97) and 0.91 (95% confidence interval 0.88 to 0.93), respectively. It should be noted that there are differences in measurements between observers, particularly regarding the few cases of cysts with large volumes (see Fig. 4). Only 31% of cysts (47/150) were large enough to have a direct relationship with the extrinsic ligaments, particularly the dorsal intercarpal ligament (DIC) and radiocarpal ligament (RCD) (see Fig. 2) corresponding to type 3 and 4 cysts according to the classification proposed by Guérini [8] (type 2, 3, and 4 in modified classification, see Table 2). When applying the modified classification, our inter-observer agreement was 0.76 (95% confidence interval 0.67 to 0.85). Among the small cysts isolated on DCSS (*n* = 103/150), the pedicle was not visible in 31 patients (modified classification 1a *n* = 31/150, see Table 2).

**Table 2:** Descriptive statistics for the study population and the subgroup with cysts

	Total population *n* = 295	Population with cysts *n* = 150
Age	39.6 (10 to 92)	38.7 (15 to 75)
Sex		
Male	54.6% *n* = 161	41.3% *n* = 62
Female	45.4% *n* = 134	58.7% *n* = 88
Hand dominance	Right 88.4% *n* = 261	Right 88% *n* = 132
Affected hand	Right 50.5% *n* = 149; Left 49.5% *n* = 146	Right 50.7% *n* = 76; Left 49.3% *n* = 74
Acute trauma	70.2% *n* = 207	50.6% *n* = 76
Injury SL	20% *n* = 59	26% *n* = 39
SL dorsal pain	22.4% *n* = 66	28% *n* = 42
Occupation		
Architecture and engineering	2.0% *n* = 6	3.3% *n* = 5
Arts design sports and media	2.4% *n* = 7	2.7% *n* = 4
Grounds cleaning and maintenance	2.4% *n* = 7	3.3% *n* = 5
Business and financial operations	0.3% *n* = 1	0.7% *n* = 1
Computer and mathematical	3.4% *n* = 10	4.7% *n* = 7
Construction and extraction	5.1% *n* = 15	6.7% *n* = 10
Education, training, and library	16.9% *n* = 50	16.7% *n* = 25
Farming fishing and forestry	1.4% *n* = 4	1.3% *n* = 2
Food preparation and serving related	7.8% *n* = 23	9.3% *n* = 14
Healthcare support	10.2% *n* = 30	12% *n* = 18
Homemaker	2.0% *n* = 6	2.0% *n* = 3
Installation maintenance and repair	5.8% *n* = 17	4.7% *n* = 7
Legal	1.0% *n* = 3	0.7% *n* = 1
Management	2.4% *n* = 7	0.7% *n* = 1
Not reported/unemployed	11.5% *n* = 34	9.3% *n* = 14
Office and administration	9.8% *n* = 29	8.7% *n* = 13
Personal care and service	3.1% *n* = 9	2.7% *n* = 4
Production	1.4% *n* = 4	0.7% *n* = 1
Protective service	1.7% *n* = 5	2.0% *n* = 3
Retired	4.7% *n* = 14	1.3% *n* = 2
Sales and related	1.7% *n* = 5	2.7% *n* = 4
Transportation and material moving	3.1% *n* = 9	4.0% *n* = 6
	**Classifications cysts in the population** ***n*** = 150	
	Guérini [8]	Modified (see “Discussion”)
	1: on the surface of SL 41.3% *n* = 62	1b: in DCSS with visible pedicle 48% *n* = 72 1a: in DCSS with no visible pedicle visible 20.7% *n* = 31
	2: within the SL 27.3% *n* = 41	2: in DCSS with extension under DIC 10% *n* = 15
	3: passing under the ST 10% *n* = 15	3: in DCSS extending dorsally to RCD 12% *n* = 18
	4: superficial to RCD and ST 21.4% *n* = 32	4: Complex cyst originating in DCSS with extension under DIC and dorsally to RCD 9.3% *n* = 14

**Fig. 5:**
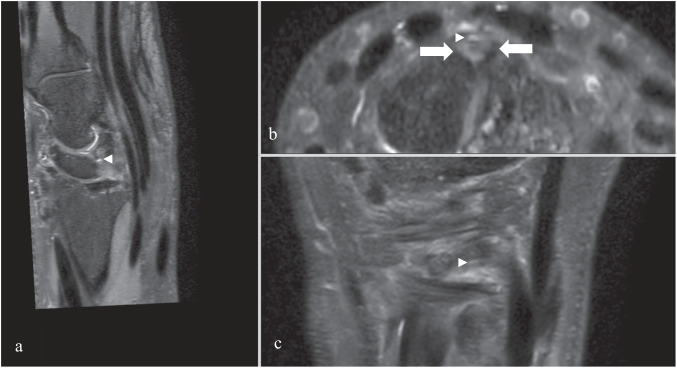
3D DP SPAIR images in MPR mode with a partial tear of the dorsal SL with a cyst next to the stumps of the ligament. **a** Sagittal plane; **b** axial plane; **c** coronal plane. Arrow = stumps of SL ligament; arrowhead = cyst

Twenty-eight percent (42/150) of patients had specific SL (scapholunate) dorsal pain, 39 showed injury to the SL ligament (palmar and/or intermediary and always associated with a partial or complete dorsal lesion; see Fig. 5), 30 of who were found to have cyst communication with the joint.

The minimum distance between the cyst wall and the PIN was assessed to be 3.2 mm on average (minimum: 0; maximum: 8.6 mm). No significant difference in job distribution was found between the total population and that with cysts (see Table 2).

Correlation tests were carried out between the various epidemiological variables collected (see Table 3). In view of the high inter-observer reliability, correlation tests were calculated according to the measurements of the most experienced observer.

**Table 3:** Correlation tests between variables in the population with the dorsal cyst

*Variable Y*	Volume ganglion observer 1 in mm^3^	Distance to PIN in mm	Trauma yes/no	Dominant hand	Guérini Classification
*Variable X*	Pain SL yes/no	Pain SL yes/no	Volume ganglion observer 1 in mm^3^	Affected hand	Pain SL yes/no
*Correlation coefficient r*	0.03730	−0.2415	−0.02604	0.1691	0.2466
*Significance level*	*p* = 0.6504	*p* = 0.0029	*p* = 0.7518	*p* = 0.0386	*p* = 0.0024
*95% Confidence interval for r*	−0.1237 to 0.1964	−0.3868 to −0.08451	−0.1855 to 0.1348	0.009041 to 0.3206	0.08987 to 0.3914

## Discussion

Several factors may explain the high prevalence (50.8%; *n* = 150/295) of cysts found in our population. We used a 3-T MRI with a high magnetic field for increased spatial resolution and a slice thickness on the 3D DP spectral attenuated inversion recovery (SPAIR) sequence of around 0.45 mm, allowing better detection and accurate assessment of very small structures in the millimeter range. Our inter-observer agreement was excellent for detection, volume, and major cyst axis measurements. These findings are better than those reported in the literature [10] based on 1.89 T MRI. We attribute these results to the technical improvements brought by 3-T imaging and 3D image reconstructions with MPR mode. As Lowden et al [10] suggest, another possible explanation for our high prevalence is the “Hawthorne effect”, whereby observers’ behavior changes because of participating in a study. An observer may have searched more intensely for cysts or interpreted the results as showing cysts because of their participation in the study. Nevertheless, the high inter-observer agreement reinforces our belief that observer bias is not the main cause of our high prevalence.

Epidemiological data for our population with cysts are in line with the literature [7, 11–13] with an average age of 38.7 years (aged 15 to 75 years) and a sex ratio of 0.6 in favor of females. 88% of our patients were right-handed and the distribution of affected hands was equitable between the two sides. The Pearson correlation test found no significant correlation between the hand affected by the cyst and the dominant hand (*r* = 0.1691, *p* < 0.05 see Table 3).

As previously mentioned, most cysts studied were small and discrepancies in measurements between observers were noted for large cysts (see Fig. 4). We explain these differences by the complexity of the anatomy of large cysts. Small cysts are generally perfectly spherical or ellipsoidal and their measurements are more easily reproducible between observers. Large cysts are generally multiloculated with septations and their anatomy is more difficult to assess even when reconstructing in 3 planes with the MPR mode. We cannot conclude statistically about the existence of a correlation between volume ganglion and the existence of acute trauma (*r* = −0.02604; *p* = 0.75).

Some authors report the need for a pre-existing underlying scapholunate pathology in the pathophysiology of dorsal cyst development [7]. We found an SL lesion in 39 cases (26%), and although we did not assess the existence of associated scapholunate instability, the number of SL lesions found is close to the 30% of scapholunate instability found in certain arthroscopic studies [7]. However, we cannot draw any conclusions on this point.

The origin of pain in most patients with dorsal cysts is controversial, and several hypotheses have been put forward in the literature, including the possibility of irritation of the dorsal sensory branches of the PIN [2, 7, 14]. We therefore measured the minimum distance between the PIN and the cystic wall (See results). A weak negative correlation was found between distance to PIN and dorsal SL pain (*r* = −0.2415; *p* < 0.05), a weak positive correlation between Guérini’s classification and dorsal SL pain (*r* = 0.2466; *p* < 0.05), and we cannot conclude statistically about the existence of a correlation between volume ganglion and dorsal SL pain (*r* = 0.03730; *p* = 0.65). Although Guérini’s classification is out of date in relation to anatomical knowledge and the discovery of the DCSS, it indirectly reflects cystic volume. All confirm that the origin of pain is complex and probably multifactorial [7], irritation of the PIN and cystic volume alone cannot explain the symptoms.

In the absence of gadolinium injection, we were unable to assess the contrast uptake of the cystic walls and the importance of peri-cystic inflammation in the origin of the pain [7].

Given their benign origin and a spontaneous regression rate of around 50% [2], conservative treatment is the treatment of first choice [3, 15, 16]. Simple aspiration of the cyst may be proposed if it persists, although the recurrence rate is around 59% [17]. Needling techniques [2, 18–20], infiltration with hyaluronidase, or the use of intra-cyst corticoids [18] have not shown better results than simple infiltration.

Surgical excision by open surgery or arthroscopy may be proposed in the event of recurrence, with rates of recurrence and complications that vary in the literature, estimated at 21% of recurrence and 14% of complications in open surgery and, respectively, 6% of recurrence and 4% of complications in arthroscopic surgery [3].

**Fig. 6:**
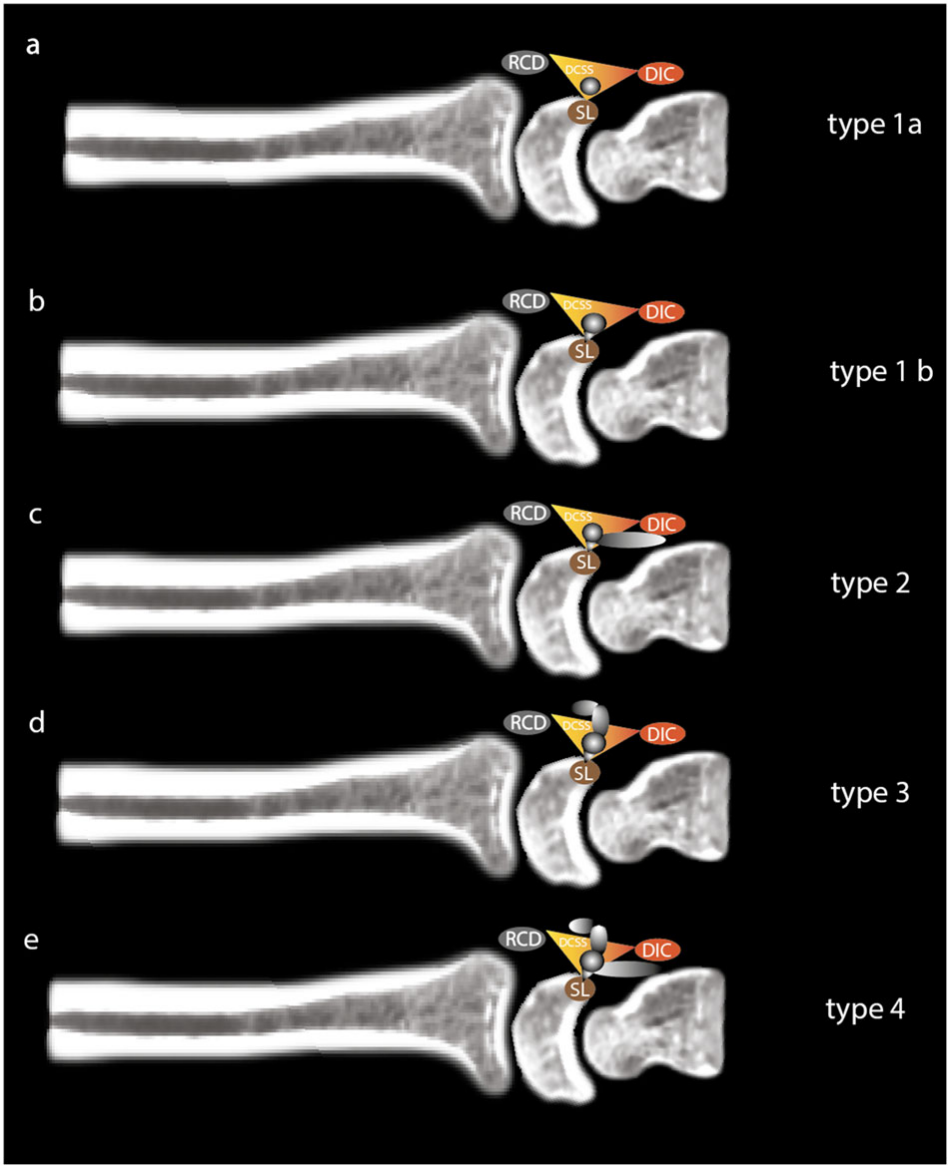
Classification of mucoid cysts of the dorsal aspect of the wrist at the expense of the DCSS and the SL ligament. **a** Type 1a: Cyst originating in the DCSS with no visible pedicle in the SL; **b** type 1b: Cyst originating in the DCSS with visible pedicle in the SL; **c** type 2: Cyst originating in the DCSS with extension under the DIC; **d** type 3: Cyst originating in DCSS extending dorsally to RCD; **e** type 4: Complex cyst originating in DCSS with extension under DIC and dorsally to RCD. DIC, dorsal intercarpal ligament; RCD, radio-carpal ligament; DCSS, dorsal capsular scapholunate septum; SL, scapholunate ligament

We would draw attention to the importance of preoperative MRI in these cases. The use of high-field MRI should provide a precise description of the anatomy of the cyst, its relationship with neighboring structures, in particular the DCSS and DIC, which must be preserved during surgery, and above all the pedicle at the origin of the cyst. The latter should be resected at the same time as the cyst [3]. An adaptation of Guérini’s classification, considering current anatomical knowledge incorporating DCSS, SL pedicle recognition, and location of the cyst in relation to the DIC, would probably be beneficial for anatomical assessment prior to any surgical management, which is why we are proposing a new radiological classification (see Fig. 6). Inter-observer agreement when using this classification was substantial. Using this new classification, we distinguished between very small cysts all located exclusively in the DCSS for which we visualized the pedicle (1b) and those for which the pedicle was not visualized (1a) around 70% *n* = 72/103 and 30% *n* = 31/103, respectively. This systematic description of whether or not the pedicle has been visualized by the radiologist using this new classification is therefore essential information to be provided to the surgeon prior to any surgical management, the aim being to complement without replacing arthroscopic observation. In addition, particular attention must be paid to the integrity of the SL and the DCSS, whose lesions will modify the surgical management technique with the addition of a capsulo-ligament repair [21].

Our study has several limitations. We studied a symptomatic population, which could have had a positive influence on the prevalence of cysts in our population. Furthermore, our study was not designed to provide recommendations for therapeutic management and no follow-up of cysts was carried out.

## Conclusion

Dorsal mucoid cyst of the wrist is a benign condition relatively common in the symptomatic population, affecting mostly women between 30 and 40 years of age, associated in 20 to 30% of cases with injury to the SL. MRI is the modality of choice for anatomical studies of this condition, with an excellent inter-observer agreement for detection and volumetric assessment. The technical improvement provided by high-field MRI should encourage the radiologist to accurately describe the anatomical relationships, size, and visibility of the pedicle, using appropriate classification, as this information is essential for preoperative assessment.

## Abbreviations

DCSS: Dorsal capsular scapholunate septum
DIC: Dorsal intercarpal ligament
MPR: Multiplanar reconstruction
MRI: Magnetic resonance imaging
PD: Proton density
PIN: Posterior interosseous nerve
RCD: Radiocarpal ligament
SL: Scapholunate ligament
SPAIR: Spectral attenuated inversion recovery
TSE: Turbo spin echo
